# Protopanaxatriol Inhibits *Listeria monocytogenes* Infection Through a Multi-Target Virulence Suppression Strategy

**DOI:** 10.3390/ijms27219745

**Published:** 2026-10-30

**Authors:** Junlu Liu, Jiahui Lu, Lanqi Liu, Rui Liu, Biyu Lan, Yingxin Qi, Decheng Yang, Hao Li, Guizhen Wang

**Affiliations:** College of Biological and Food Engineering, Jilin Engineering Normal University, Changchun 130052, China; liujunlu@stu.jlenu.edu.cn (J.L.); lujiahui@stu.jlenu.edu.cn (J.L.); liulanqi@stu.jlenu.edu.cn (L.L.); liurui@stu.jlenu.edu.cn (R.L.); lanbiyu@stu.jlenu.edu.cn (B.L.); qiyingxin@jlenu.edu.cn (Y.Q.); ccydc2005@163.com (D.Y.); lihao@jlenu.edu.cn (H.L.)

**Keywords:** protopanaxatriol, *L. monocytogenes*, internalins, biofilm, anti-infection

## Abstract

*Listeria monocytogenes* (LM) infection relies on the synergistic effects of multiple virulence factors, including but not limited to flagella-mediated bacterial motility, listeriolysin O (LLO)-mediated immune evasion and inflammatory response, multiple virulence proteins anchored to the cell wall by SortaseA (SrtA), and internalin-mediated adhesion and invasion. These virulence factors are critical potential targets for developing anti-LM infection inhibitors. This study found that protopanaxatriol (ptt) significantly inhibited the adhesion and invasion of LM into epithelial cells potentially by interfering with internalin A/InlB (InlA/InlB)-mediated host–cell interactions and reducing the transcription of *inlA* and *inlB.* InlA was anchored to the cell wall by SrtA, and in silico analysis predicted that ptt may interact with key active residues of SrtA. Additionally, ptt was predicted to interact with LLO and inhibited its oligomer formation, resulting in a loss of its pore-forming activity. Flagella, SrtA, and LLO all affect bacterial biofilm formation; ptt inhibited the formation of biofilms and disrupted mature biofilm without affecting the growth of LM. Finally, ptt protected *Galleria mellonella* from LLO and LM by increasing survival and alleviating tissue damage. These results indicate that ptt shows excellent potential for the prevention and control of LM infection.

## 1. Introduction

*Listeria monocytogenes* (LM) is a foodborne pathogen widely distributed in nature and a causative agent of zoonotic diseases [1]. The bacterium can survive under low-temperature conditions [2]. Consumption of undercooked food poses a risk of infection, leading to human illness [3]. The primary clinical manifestations include bacteremia [4], meningitis [5], bacterial peritonitis [6], spontaneous abortion, and fetal demise [7]. As an intracellular bacterium [8], LM preferentially infects immunocompromised populations [9], such as the elderly and neonates [8]; however, the inappropriate use of antibiotics has led to the emergence of resistance during clinical treatment [10], making the development of anti-LM agents a matter of utmost urgency. In animals, LM primarily infects livestock through the fecal–oral route [11], presenting clinical symptoms similar to those observed in humans [12]. Due to its epidemiological characteristics of low incidence but high mortality [13], this pathogen has been designated as a key target for food safety surveillance by numerous countries [2]. Therefore, developing natural anti-LM infection inhibitors is important for food safety, the livestock and poultry industry, and human health.

During cellular invasion and subsequent toxicity of LM, LLO plays an essential role as the most critical virulence factor [14]. LLO is a cholesterol-dependent cytolysin (CDC) encoded by the *hly* gene [15]. It primarily functions during LM infection of phagocytes; upon phagocytosis of LM by host cells, LLO specifically recognizes cholesterol on the phagosomal membrane and oligomerizes to form a pre-pore complex. Concurrently, conformational changes in LLO molecules drive the insertion of the oligomer into the membrane, creating a pore that enables bacterial escape into the cytoplasm [15,16]. Furthermore, LLO significantly regulates LM biofilm formation; studies have demonstrated that deletion of the *hly* gene severely impairs biofilm formation, leading to reduced bacterial stability and mitigated pathogenicity [17,18]. Consequently, LLO serves as a vital target for the development of anti-LM inhibitors. It has also been confirmed that several natural products, including phloretin [19], luteolin [20], morin [21], and kaempferol [22], counteract LM infection by targeting LLO activity.

Internalins serve as a “molecular key” for LM to invade non-phagocytic cells, among which InlA [23] and InlB [24] play pivotal roles in this process. InlA is anchored to the cell wall via SrtA-mediated recognition of its LPXTG motif [25], thereby targeting host E-cadherin to mediate bacterial adhesion to the cell surface [26]. InlB, on the other hand, recognizes and activates the receptor tyrosine kinase Met (c-Met) [27], exerting a critical function; it is anchored to the cell wall through non-covalent interactions, thereby facilitating LM invasion into host cells. Therefore, internalins are also regarded as potential therapeutic targets for LM. To accelerate the discovery of inhibitors against such virulence targets, in recent years, structure-based computational approaches, particularly molecular docking combined with molecular dynamics (MD) simulations, have been extensively applied in antibacterial drug discovery, enabling the rational identification and mechanistic characterization of inhibitors targeting bacterial virulence factors and other essential proteins [28]. Notably, such in silico strategies have also been successfully employed to identify novel inhibitors against the virulence regulators of LM [29].

Extracted from ginseng, protopanaxatriol (ptt) is a natural active compound [30] that has been reported to effectively treat Alzheimer’s disease (AD)-related cognitive decline [31] and reduce cardiac hypertrophy [32]. It also plays a role in inhibiting the progression of colon cancer [33]. Natural products have become potent therapies against bacterial infections and remain an important source for the discovery of novel antibacterial agents [34], showing great potential for development of novel anti-infective agents. Since preliminary screening demonstrated its excellent therapeutic effects, this study delves into the underlying therapeutic mechanisms of ptt. This research not only opens up new avenues for tackling the global challenge of antimicrobial resistance but also provides fresh solutions for controlling LM contamination in food and managing clinical infections.

## 2. Results

### 2.1. Analysis of the Results

#### 2.1.1. Ptt Inhibits the Adhesion and Invasion of LM into Host Cells by Interfering with InlA and InlB and Suppressing Their Transcription

**Figure 1:**
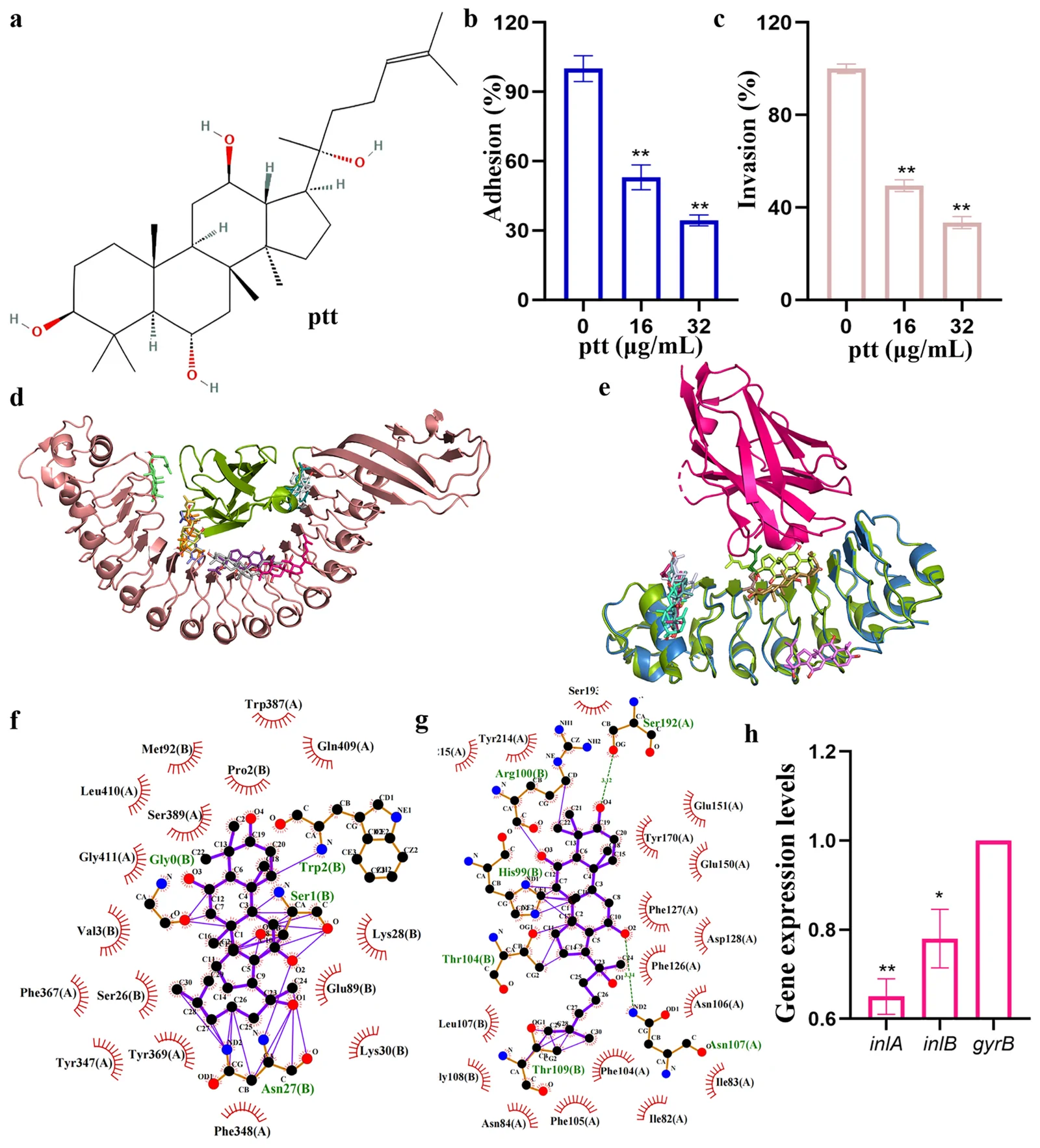
Ptt inhibits the adhesion and invasion of LM into host cells by interfering with InlA and InlB and suppressing their transcription. (**a**) The molecular structure of ptt. (**b**,**c**) The adhesion and invasion of LM cells treated with different concentrations of ptt. HeLa cells were co-incubated with LM and different concentrations of ptt, lysed, and plated for bacterial enumeration. The invasion test must be treated with gentamicin on this basis to kill extracellular bacteria. (**d**,**e**) Potential binding modes of ptt with InlA/InlB. The docking was predicted using AutoDock Vina 1.2.7. (**f**,**g**) Potential interaction sites between InlA/InlB and ptt. The binding sites were analyzed based on LigPlus v2.2.9. (**h**) Ptt suppresses the transcription of LM internalin genes (*inlA/inlB*). After LM co-incubation with ptt, RNA was extracted for reverse transcription, and then real-time quantitative PCR (qPCR) was performed to detect the expression of *inlA* and *inlB*. Data are presented as means ± standard deviations (SDs, n = 3); * *p* < 0.05 and ** *p* < 0.01.

The molecular structure of ptt is shown in Figure 1a. In the cell-based assays, the percentage of adhesion decreased to approximately 57% and 32% (Figure 1b), while the percentage of invasion decreased to approximately 50% and 35% (Figure 1c), respectively, when the samples were treated with 16 or 32 μg/mL ptt.

It has been demonstrated that InlA and InlB mainly mediate the adhesion and the invasion of LM. The crystal structures of InlA and its host receptor E-cadherin have been reported, and the interactive interface analysis showed that many residues are involved in the interaction, including Trp387, Tyr347, and Gln409, among others (Figure S1a,b). Similarly, the residues involved in the interaction between InlB and its host receptor are Phe104, Phe105, Phe127, etc. (Figure S2a,b). Here, molecular docking was performed to predict the potential binding of ptt to InlA/InlB. The results suggest that ptt may bind to the interaction interface of InlA/InlB and their host receptors (Figure 1d,e). The residues predicted to interact with ptt included Trp387, Tyr347, Gln409, Phe104, Phe105, and Phe127 (Figure 1f,g and Figure S3a,b).

To evaluate the effect of ptt on the transcription of *inlA* and *inlB*, the expression levels of these genes were determined. As shown in Figure 1h, the relative expression level of *inlA* was significantly reduced after ptt treatment, and the relative expression level of *inlB* was also significantly downregulated. These results indicate that ptt inhibits the adhesion and invasion of LM into host cells, which may be associated with the reduced transcription of *inlA* and *inlB*. Molecular docking further suggested that ptt may interfere with the interaction interfaces between InlA/InlB and their host receptors, providing a potential additional mechanism.

#### 2.1.2. Ptt Inhibits the Biological Activity of SrtA by Interacting with Critical Active Residues

**Figure 2:**
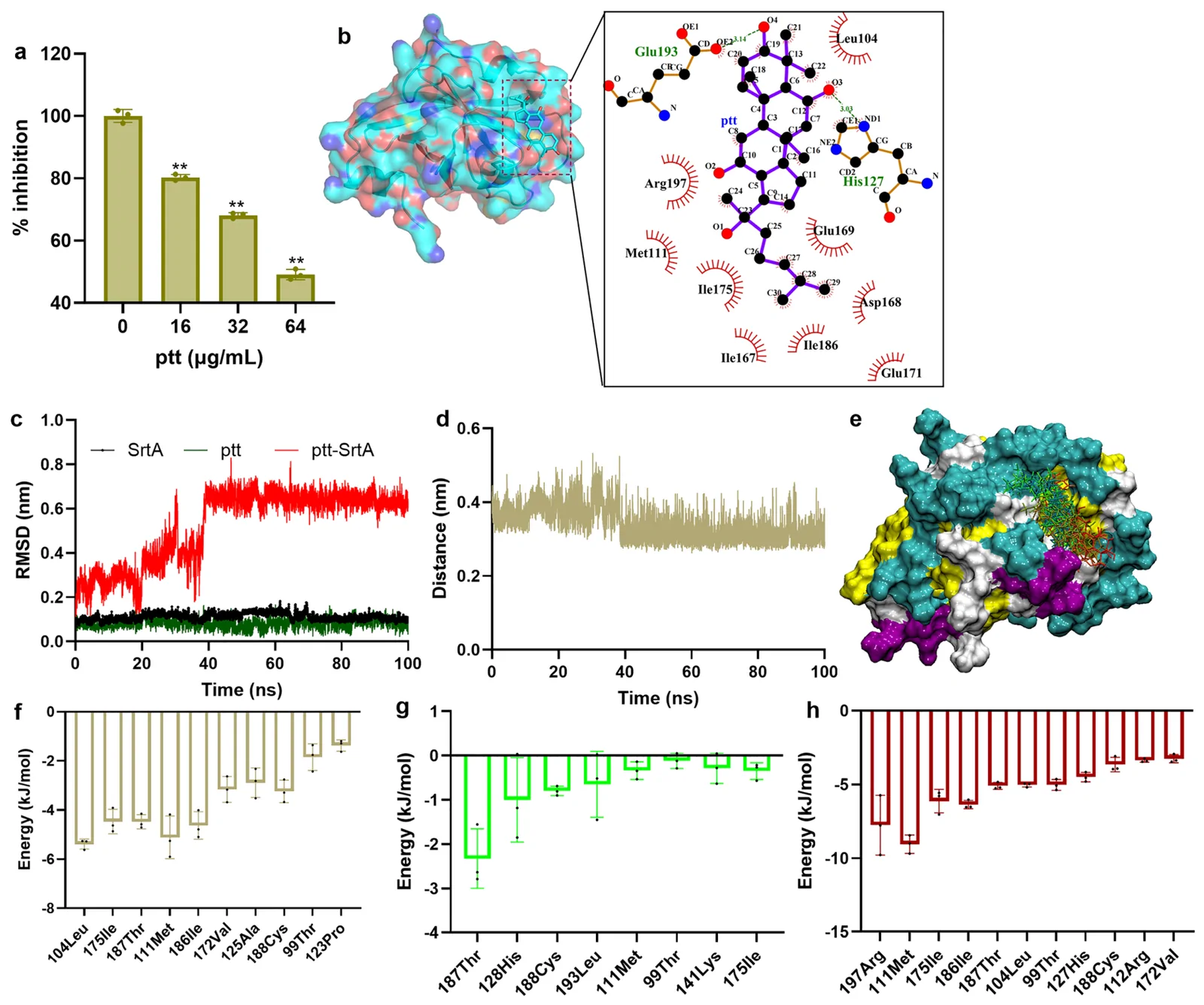
Ptt inhibits the biological activity of SrtA by interacting with critical active residues. (**a**) Dose-dependent inhibitory effect of ptt on SrtA activity. Purified SrtA protein was incubated with different concentrations of ptt at 37 °C for 30 min, followed by addition of the fluorogenic substrate. Fluorescence signals were recorded after incubation in the dark for 1 h. (**b**) The binding mode of ptt and SrtA and potential binding sites. The binding modes were predicted using AutoDock Vina 1.2.7. (**c**) RMSD profiles of SrtA and the ptt-SrtA complex during 100 ns MD simulation. (**d**) Distance variations between ptt and SrtA during the MD simulation. (**e**) Relative position of ptt within the SrtA binding pocket at different simulation times. (**f**–**h**) Weak interactions between ptt and critical SrtA. The RMSD and distance data were analyzed based on GROMACS software (version 2020.6), the relative position was generated using VMD 1.9.3, and the binding free energies were analyzed using the Molecular Mechanics-Poisson-Boltzmann Surface Area (MMPBSA) program. Data are presented as means ± SDs (n = 3); ** *p* < 0.01.

As a surface protein anchored to the cell wall, InlA is covalently anchored to the bacterial cell wall by SrtA. Here, we explored the inhibitory effect of ptt against SrtA function. It was found that ptt exhibited a significant dose-dependent inhibitory effect on SrtA activity, with the inhibition rate gradually increasing from 16 to 64 μg/mL (Figure 2a). Molecular docking predicted that ptt could occupy the active pocket of SrtA and potentially interact with the key catalytic residues His127, Arg197, and Cys188 (Figure 2b). The stability of this predicted binding mode was further evaluated by MD simulations; the complex remained stable throughout the entire simulation process, with the complex root-mean-square deviation (RMSD) stabilized at approximately 0.6 nm after 40 ns (Figure 2c). The distance between ptt and SrtA remained consistently below 0.4 nm (Figure 2d); ptt never dissociated from the active pocket during the entire simulation (Figure 2e). The distance and positional analyses support the stability of the predicted binding mode. Residue-based binding-free-energy decomposition showed that Leu104, Met111, and Ile186 contributed more to the total binding free energy (Figure 2f); Thr187, His128, and Cys188 provided more electrostatic interactions (Figure 2g); and Arg197, Met111, His127, and Cys188 contributed more to the van der Waals interactions (Figure 2h). Moreover, no co-crystal structure of LM SrtA with its substrate has been reported to date. Given the high homology between LM SrtA and *Staphylococcus aureus* SrtA (Sa-SrtA), for which the co-crystal structure with its natural substrate peptide LPETG has been reported, we aligned the predicted LM SrtA–ptt complex with the Sa-SrtA–LPETG structure. As shown in Figure S4, the binding position of ptt overlapped with the LPETG binding site, indicating that ptt may occupy the substrate-binding pocket and thereby hinder the recognition of LPXTG-motif substrates by SrtA. These results suggest that ptt may interact with the active pocket of SrtA and potentially interact with key catalytic residues, thereby inhibiting the biological activity of SrtA.

#### 2.1.3. Ptt Effectively Inhibits the Hemolytic Activity of LLO and LM Culture Supernatant

**Figure 3:**
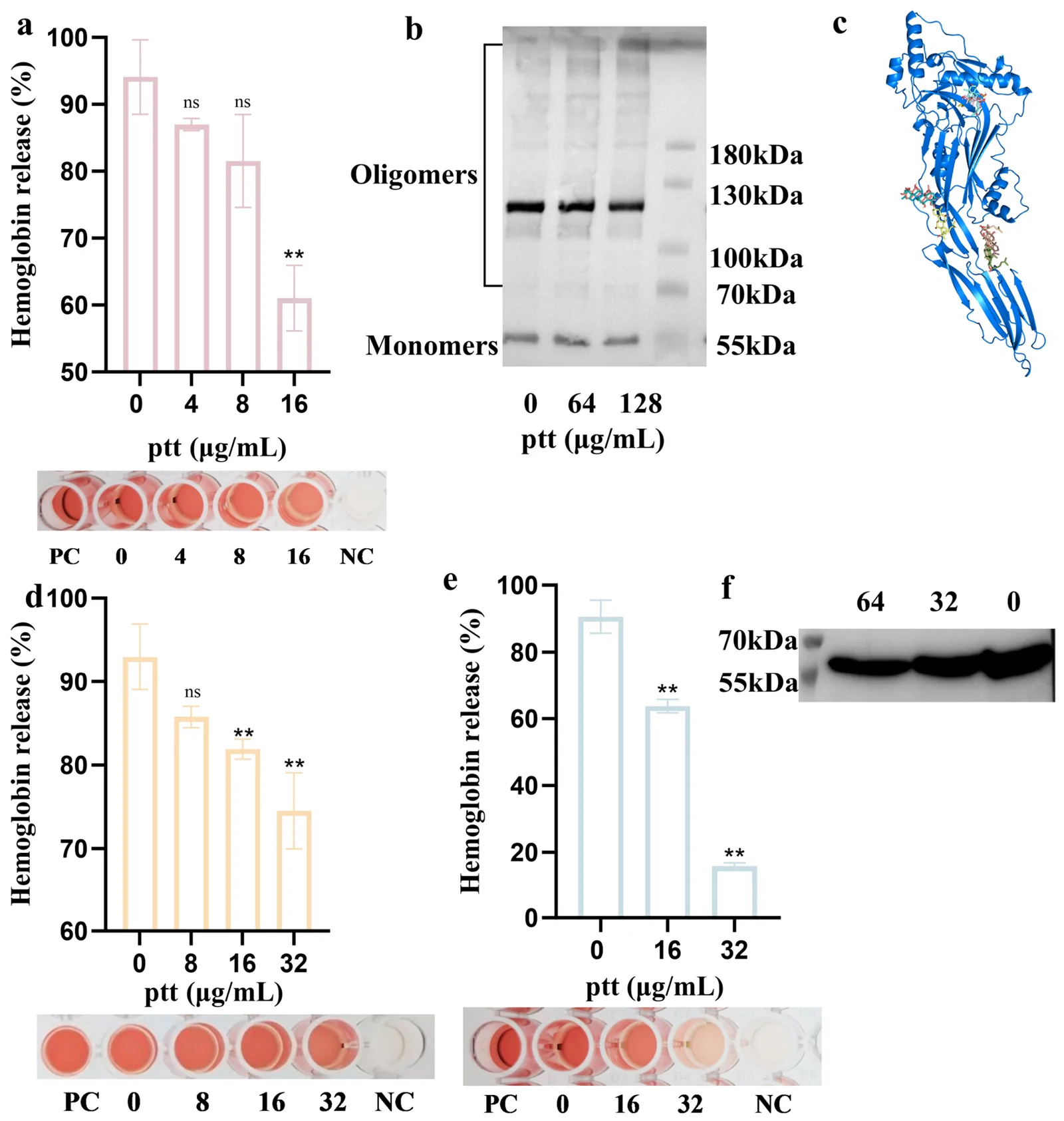
Ptt effectively inhibits the hemolytic activity of LLO and LM culture supernatant. (**a**) Hemolytic activity of LLO treated with ptt against sterile defibrinated sheep blood. The purified LLO was incubated with ptt at different concentrations and then with sterile defibrinated sheep blood. Absorbance at 543 nm (Abs543) was measured to determine the hemolysis condition. (**b**) The amounts of LLO monomers and oligomers formed after treatment with different concentrations of ptt. LLO was incubated with different concentrations of ptt, and oligomerization was induced by the addition of sheep red blood cells. Oligomer and monomer formation was analyzed by Western blotting. (**c**) Potential binding mode between ptt and LLO. (**d**) The influence of ptt on the hemolytic activity of LM culture supernatants. LM supernatants were incubated with different concentrations of ptt, and then sterile defibrinated sheep red blood cells were added and incubated for 10 min. The supernatants were collected, and Abs543 was measured. (**e**) Hemolytic activity of LM culture supernatants that co-cultured with ptt. After overnight co-culture of LM with different concentrations of ptt, the supernatants were collected and mixed with sterile defibrinated sheep blood, and Abs543 was measured. (**f**) Ptt inhibits the secretion of LLO. The levels of LLO in culture supernatants following overnight co-culture with different concentrations of ptt were assessed by Western blotting. Data are presented as means ± SDs (n = 3); ns, not significant; ** *p* < 0.01.

To investigate the inhibitory effect of ptt against LLO and LM culture supernatant hemolytic activity, hemolysis assays were conducted. The results showed that when purified LLO protein was co-incubated with different concentrations of ptt, the hemoglobin release rates were 94.10%, 87.00%, 81.53%, and 61.03% (Figure 3a). The oligomer is the prerequisite for LLO to exert its hemolytic activity. Here, we confirmed that ptt treatment reduced the formation of LLO oligomers (Figure 3b and Figure S5a), suggesting that ptt may interact with LLO. Molecular docking further predicted three potential binding modes of ptt on LLO (Figure 3c). Notably, since all domains of LLO contribute to oligomer formation, blind docking was adopted in this study; in addition to the domain 3/domain 4 junction, other potential binding sites of ptt were identified, suggesting that ptt may inhibit LLO through mechanisms distinct from those of amentoflavone and atractylodin. These binding modes blocked the formation of the oligomers. The hemolytic activity of LM culture supernatants treated with 0, 8, 16, and 32 μg/mL ptt was significantly reduced, with values of 92.99%, 85.77%, 81.92%, and 74.52%, respectively (Figure 3d). The hemolysis rate of LM culture supernatant without ptt treatment was 90.63%, whereas co-incubation with 16 μg/mL and 32 μg/mL ptt reduced it to 63.81% and 15.90% (Figure 3e). Detection of secreted proteins in the supernatant showed that ptt treatment reduced the secretion of LLO (Figure 3f and Figure S5b). The purities of LLO and SrtA are shown in Figure S5c. To evaluate the reliability of our docking protocol, we further docked two reported LLO inhibitors, amentoflavone and atractylodin, with LLO. Both compounds were predicted to bind to the junction between domains 3 and 4 of LLO, consistent with their previously reported binding sites, and ptt was also found to bind to this pocket (Figure S6), supporting the validity of our docking results. Taken together, ptt was predicted to interact with LLO and inhibited its oligomer formation, resulting in the loss of LLO function; additionally, ptt inactivated the hemolytic activity of LM culture by reducing LLO secretion.

#### 2.1.4. Ptt Inhibits the Swimming Motility of LM by Regulating the Expression of Flagellar Genes *fliY*, *fliM*, and *flhB*

**Figure 4:**
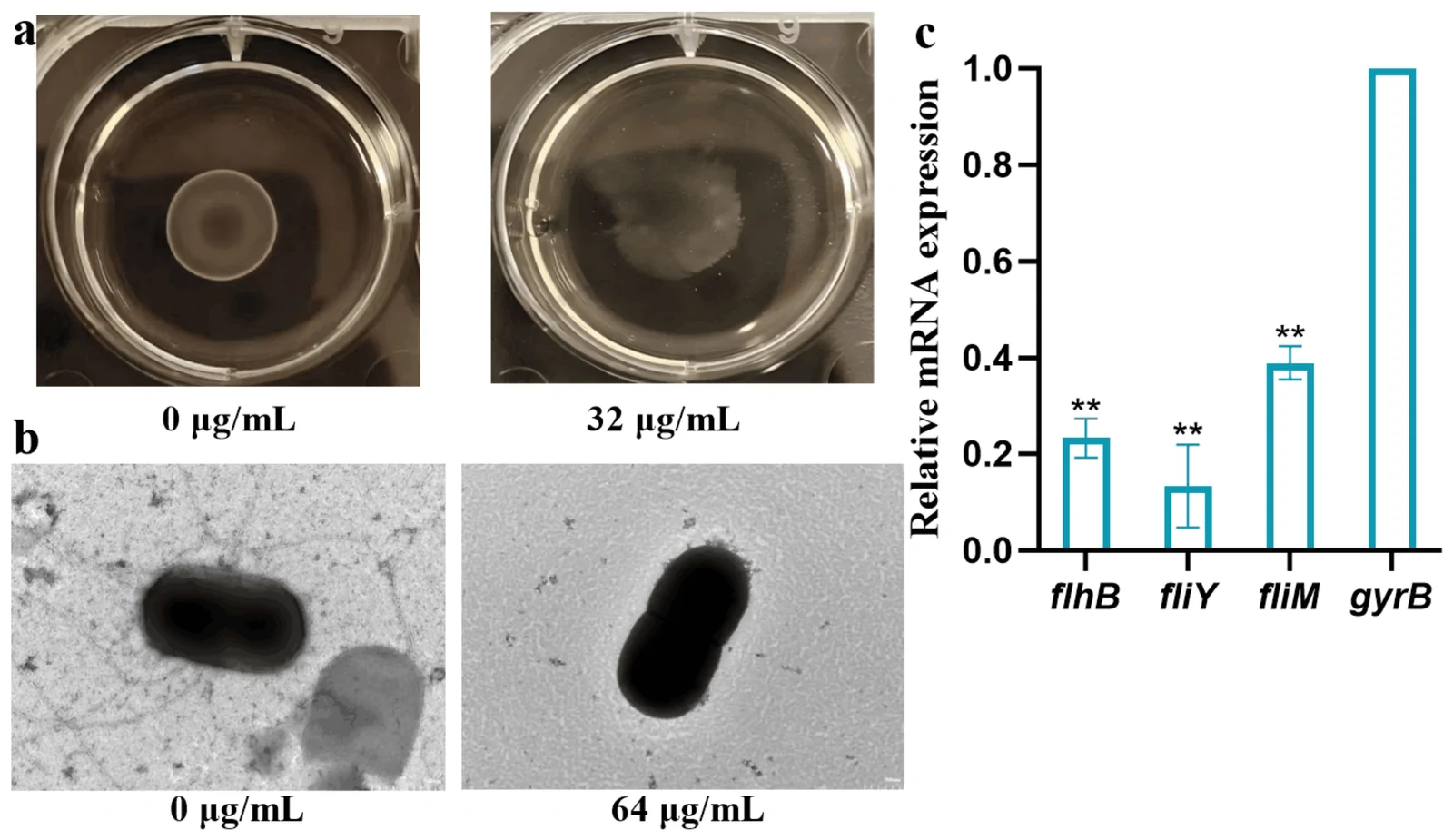
Ptt inhibits the swimming motility of LM by regulating the expression of flagellar genes *fliY*, *fliM*, and *flhB*. (**a**) The swimming motility of bacteria when treated with or without ptt. Swimming motility was determined by measuring the spreading diameter of LM cultured on semi-solid agar plates. (**b**) The flagellar formation of LM when treated with or without ptt. Flagellar morphology of LM with or without ptt treatment was visualized by transmission electron microscopy (TEM). (**c**) The expression levels of flagellar genes in bacteria after ptt treatment. LM was co-cultured with different concentrations of ptt, the bacteria were collected, and total RNA was extracted. After reverse transcription to obtain cDNA, the expression levels of the target genes were analyzed by RT-qPCR. Data are presented as means ± SDs (n = 3); ** *p* < 0.01.

Flagella play an important role in LM motility and may contribute to subsequent colonization. Experimental results demonstrated that the untreated control group exhibited distinct concentric swimming motility (Figure 4a). In contrast, in the medium treated with 32 µg/mL ptt, the shape of the concentric circles was disrupted to the extent that no clear morphology was observable(Figure 4a). Furthermore, the bacterial flagella were rarely observed after treatment with ptt (Figure 4b), indicating that ptt exerts a significant inhibitory effect on LM flagellar formation. Additionally, the mRNA expression levels of flagella-related genes were significantly downregulated after ptt treatment, with relative expression levels decreasing to approximately 0.13 for *fliY*, 0.39 for *fliM*, and 0.23 for *flhB* (Figure 4c). These data indicate that ptt significantly affects the transcriptional expression of *fliY*, *fliM*, and *flhB*, thereby inhibiting bacterial flagellar formation and consequently reducing the motility of LM.

#### 2.1.5. Ptt Disrupts the Mature Biofilm of LM and Inhibits Biofilm Formation Without Inhibiting Bacterial Growth

**Figure 5:**
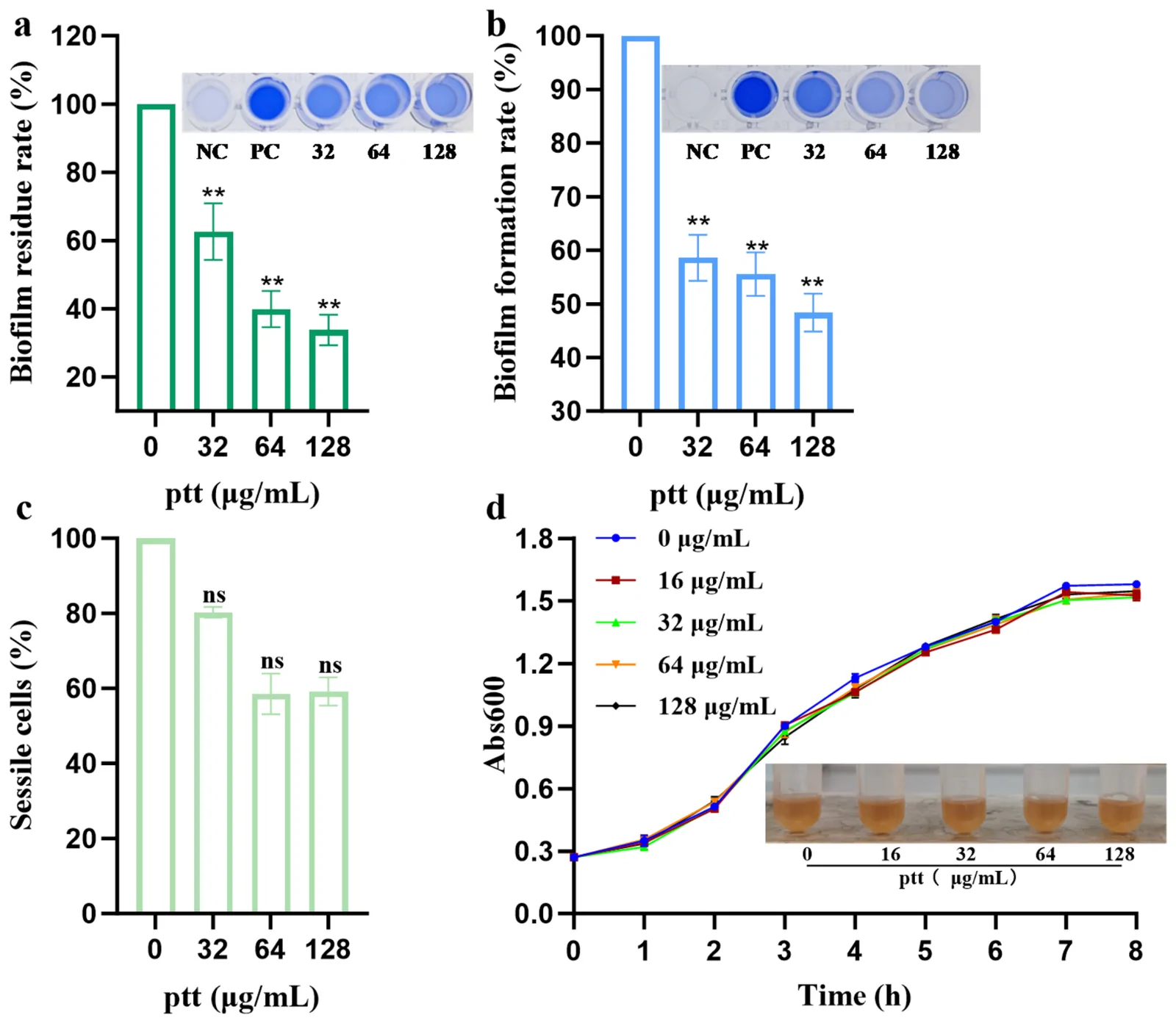
Ptt disrupts the mature biofilm of LM and inhibits biofilm formation without inhibiting bacterial growth. (**a**) The eradication effect of ptt on LM mature biofilm. LM was cultured in 96-well plates for 24 h. The planktonic bacteria were then removed, and the samples were washed and treated with sterile phosphate-buffered saline (PBS) containing different concentrations of ptt for 24 h. Residual biofilm was analyzed by crystal violet staining. (**b**) Biofilm formation of LM when treated with various concentrations of ptt. LM was co-cultured with different concentrations of ptt for 24 h. The planktonic bacteria were removed, and the samples were washed, air-dried, and stained with crystal violet. After solubilization with glacial acetic acid, Abs570 was measured. (**c**) The number of viable bacteria in LM biofilms after ptt treatment. After co-incubation of LM with ptt, bacteria in the biofilm were collected and plated for colony counting to analyze the effect of ptt on the number of viable bacteria in the biofilm. (**d**) The growth curve of LM after ptt treatment. LM was cultured in the presence of various concentrations of ptt. The Abs600 was measured every hour, and growth curves were plotted over time. Data are presented as means ± SDs (n = 3); ** *p* < 0.01, and ns, not significant.

The crystal violet staining assay results demonstrated a concentration-dependent reduction in the relative biofilm biomass of the ptt-treated groups compared to the control group (0 µg/mL), specifically, the residual rate decreased to 62.59% in the 32 µg/mL group, 39.91% in the 64 µg/mL group, and 33.80% in the 128 µg/mL group (Figure 5a), indicating that ptt exerted a significant disruptive effect on pre-formed biofilms within the tested concentration range. Consistently, the biofilm formation assay results show that ptt significantly inhibited the initial formation of biofilms at a concentration as low as 32 µg/mL; compared with the control group (0 µg/mL), biofilm formation decreased to 58.60% in the 32 μg/mL group, to 55.55% in the 64 μg/mL group, and to approximately 48.36% in the 128 μg/mL group (Figure 5b). The bacteria embedded in the biofilms were collected and enumerated by plate counting. The results indicate that treatment with different concentrations of ptt did not significantly affect the number of viable bacteria within the biofilm compared to the control (Figure 5c). Furthermore, the minimum inhibitory concentration (MIC) of ptt against LM was greater than 1024 µg/mL. Notably, the growth trends of LM remained nearly identical at different ptt concentrations (0, 16, 32, 64, and 128 µg/mL) (Figure 5d), demonstrating that ptt does not inhibit the growth of LM.

#### 2.1.6. Ptt Protects *G. mellonella* from LLO Toxicity In Vivo

**Figure 6:**
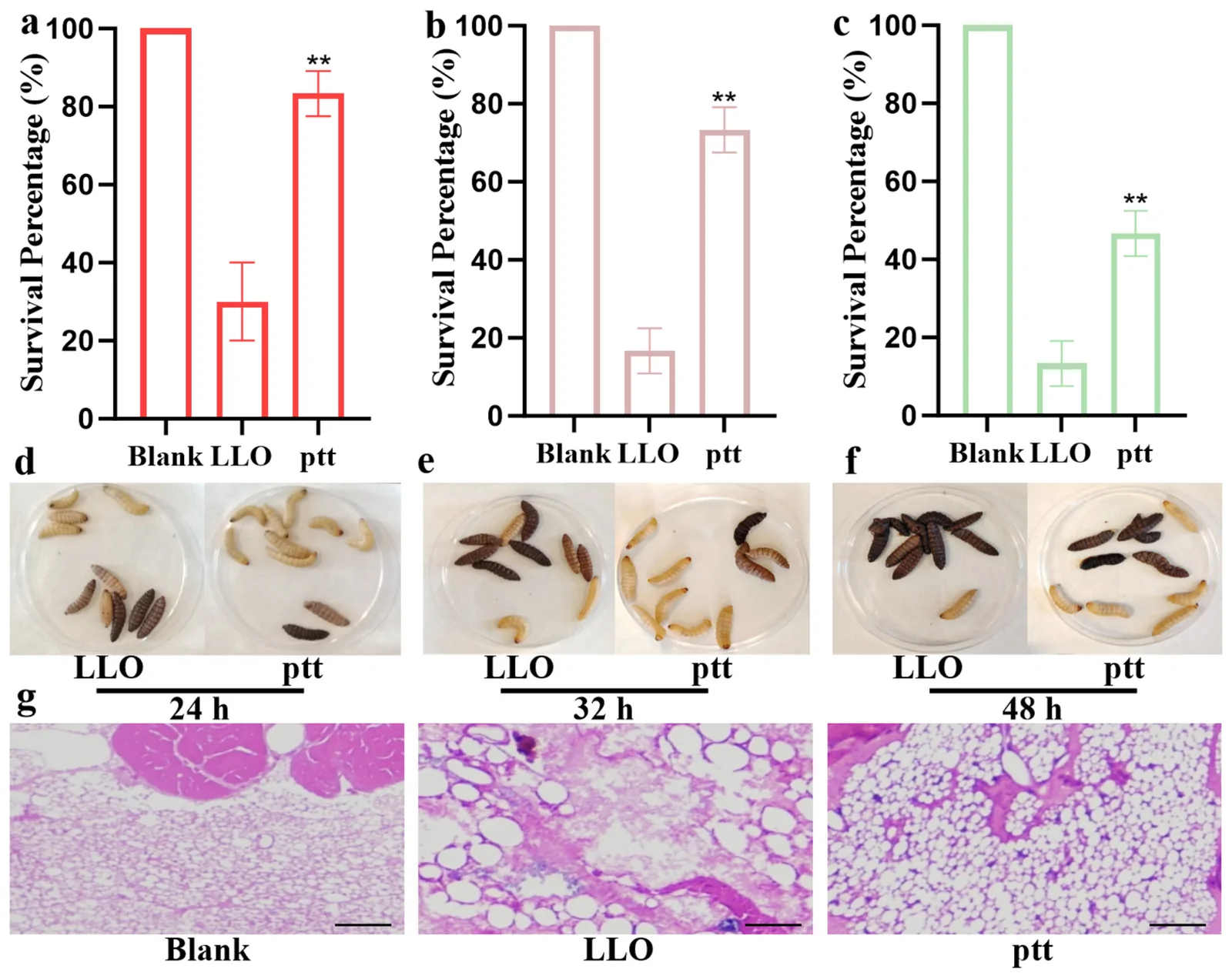
Ptt protects *G. mellonella* from LLO toxicity in vivo. (**a**–**c**) Survival rates were measured 24, 32, and 48 h after injection. LLO was co-incubated with ptt and then injected into *G. mellonella*. Larval survival was monitored at the designated time points. (Three groups of independent repeated experiments, n = 10 per group.) Data are presented as means ± SDs; ** *p* < 0.01. (**d**–**f**) Representative appearance of *G. mellonella* after LLO infection. (**g**) The tissue conditions of *G. mellonella* under different treatment methods. Histopathological changes were evaluated by hematoxylin and eosin (H&E) staining. Scale bar = 200 μm.

To verify whether ptt could still exert a protective effect against LLO toxicity in vivo, we established a *G. mellonella*-LLO model. Following LLO injection, *G. mellonella* exhibited high mortality; the 24-h survival rate was only 30.00% (Figure 6a), which further dropped to 16.67% at 32 h (Figure 6b), and the 48-h survival rate was 13.33% (Figure 6c). However, the survival rate of the ptt treatment group was significantly higher than that of the LLO-injected group, reaching 83.33% at 24 h (Figure 6a) and maintaining a high level of 73.33% at 32 h (Figure 6b). The survival rate was 46.67% after 48 h (Figure 6c). Phenotypic observations revealed that LLO infection induced significant swelling and darkening in *G. mellonella*, whereas ptt treatment reduced melanization intensity (Figure 6d–f). Histopathological examination revealed that LLO-treated *G. mellonella* exhibited severe tissue damage, characterized by disrupted tissue architecture. These pathological manifestations were markedly ameliorated following ptt treatment (Figure 6g). Collectively, these findings demonstrate that ptt can effectively antagonize LLO toxicity and significantly improve the survival rate of *G. mellonella*.

#### 2.1.7. Ptt Exerts Protective Effects on *G. mellonella* Challenged with LM

**Figure 7:**
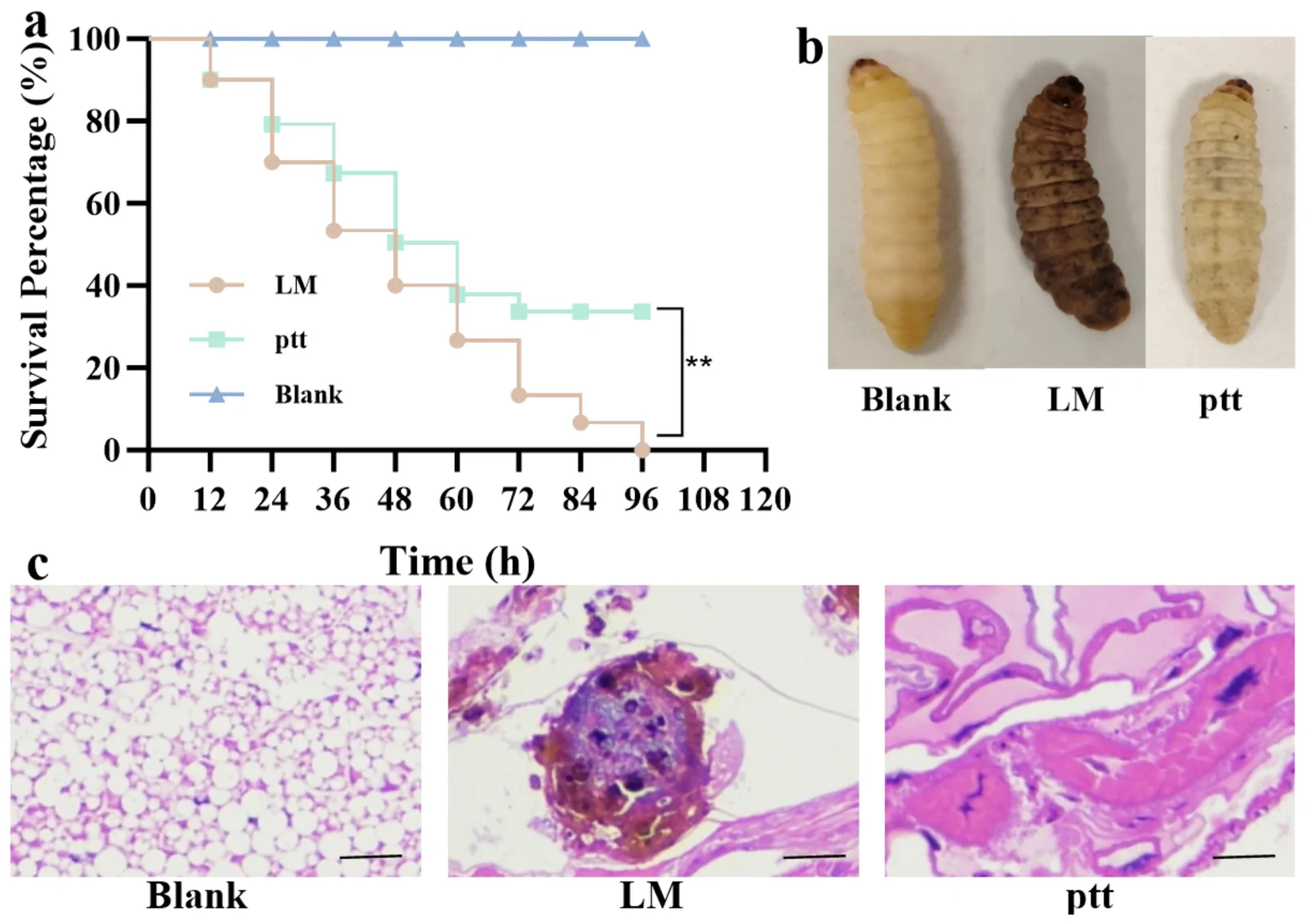
Ptt exerts protective effects on *G. mellonella* challenged with LM. (**a**) Effect of ptt on the survival rate of *G. mellonella* infected with LM. *G. mellonella* were infected with LM, followed by treatment with ptt. Larval survival was monitored at the indicated time points, and Kaplan–Meier survival curves were generated (three groups of independent repeated experiments, n = 10 per group). Statistical significance was determined by log-rank (Mantel–Cox) test; ** *p* < 0.01. (**b**) LM induces individual injury phenotypes of *G. mellonella*. (**c**) The tissue condition of *G. mellonella* after ptt treatment. This histological change was observed through the H&E staining of tissue sections. Scale bar = 200 μm.

In the LM infection model, no mortality was observed in the blank control group during the 96-h monitoring period, with 100% survival. In contrast, the infection group began to exhibit mortality 12 h post-infection, with the number of dead individuals increasing significantly over time; no survivors remained at 96 h, while for the ptt treatment group, the final survival rate was 33.66% (Figure 7a). Individuals from the blank group appeared white without spots; the LM-infected individuals were swollen and filled with melanin throughout the body, while pathological symptoms in the ptt-treated group were significantly alleviated (Figure 7b). In terms of tissue damage, the blank group did not show infectious characteristics; LM infection caused severe tissue necrosis, while ptt treatment mitigated LM-induced tissue damage (Figure 7c). These results suggest that ptt reduces the pathogenicity of LM in vivo by improving survival and relieving tissue damage.

## 3. Discussion

Currently, clinical treatment for LM infection primarily relies on antibiotics [35]. However, low-dose antibiotics exert limited efficacy against intracellular LM, whereas high-dose administration tends to induce bacterial resistance [36], thereby exacerbating the difficulty of clinical management. Consequently, identifying and developing novel antimicrobial agents represents a critical direction in combating LM infections. As a typical intracellular pathogen, the pathogenicity of LM depends predominantly on its virulence factors [11,37], which enable the bacteria to evade host immune clearance, survive, and disseminate. Therefore, inhibitors targeting these virulence factors, rather than employing a conventional bactericidal approach, constitute a pivotal research strategy [38].

Adhesion and invasion into host cells are critical initial events for intracellular LM infection [25]. InlA and InlB play pivotal roles in mediating these processes [39,40]. Although InlA- and InlB-targeting inhibitors have been identified [41], their therapeutic potential remains limited due to their single-target mode of action and an incomplete understanding of their molecular mechanisms. Moreover, current strategies mainly focus on disrupting the interactions between these virulence factors and host receptors [42,43], while approaches aimed at modulating the expression of these key virulence determinants have remained limited. In this study, we found that ptt markedly attenuates LM adhesion to and invasion into epithelial cells. The reduced expression of *inlA* and *inlB* may contribute to these effects, while molecular docking predicted that ptt could interact with regions involved in InlA–host receptor and InlB–host receptor interactions.

Following adhesion and invasion, LM establishes intracellular infection through the coordinated action of multiple virulence factors [44]. Among them, SrtA anchors more than ten LPXTG-motif-containing virulence proteins to the bacterial cell wall surface, and most internalin family proteins are attached to the cell wall through SrtA-mediated anchoring [43]. LLO, a pore-forming toxin, facilitates LM escape from host immune cell-mediated killing by disrupting the phagosomal membrane, thereby promoting intracellular survival [45]. In addition, flagella-mediated motility enables bacterial movement toward target tissues and contributes to subsequent colonization [46]. Ptt significantly downregulated the expression of the flagellar-associated genes *fliY*, *fliM*, and *flhB*, suggesting that ptt may interfere with flagellar assembly and consequently impair LM motility. The unchanged expression of *gyrB* supports its suitability as a reference gene under the experimental conditions [47].

In this study, we demonstrated that ptt was predicted to interact with key active residues of SrtA, reducing the anchoring of SrtA-dependent virulence proteins to the bacterial cell wall. Furthermore, ptt inhibited LLO oligomerization and decreased LLO secretion into the extracellular environment, consequently attenuating the hemolytic activity of both purified LLO protein and LM culture supernatants, which may impair LM escape from phagosomes. Moreover, ptt suppressed flagellar formation by downregulating the transcription of key flagellar genes, thereby weakening the swimming motility of LM.

Previous studies have demonstrated that LLO [48], SrtA [49], and flagella [50,51] are closely associated with biofilm formation. In this study, we confirmed that ptt not only inhibited the formation of LM biofilms but also disrupted pre-established mature biofilms, indicating its potential to alleviate persistent LM infections. Furthermore, by establishing in vivo infection models, we demonstrated that ptt provided systemic protective effects against both LLO-mediated toxicity and LM infection in vivo. Collectively, although several LM virulence inhibitors have been reported, most exhibit single or dual-targeting activity. Considering the complex infection process of LM and the synergistic actions of multiple virulence factors, ptt represents a promising candidate for the development of novel anti-LM strategies. However, several lines of work remain to be pursued in future studies. For instance, the interaction between ptt and its target protein could be further validated by approaches such as surface plasmon resonance (SPR), isothermal titration calorimetry (ITC), or co-crystallization. In addition, the protective effect of ptt against LM infection in vivo should be confirmed in mammalian models, and the metabolism and bioavailability of ptt in vivo need to be clarified.

## 4. Materials and Methods

### 4.1. Cells, Strains, and Culture Conditions

LM (BAA-679, also known as EGD-e, purchased from ATCC) was cultured in Brain Heart Infusion (BHI) medium at 37 °C with constant shaking at 200 revolutions per minute (rpm) until the mid-logarithmic growth phase. HeLa cells were maintained in Dulbecco’s Modified Eagle Medium (DMEM) supplemented with 10% fetal bovine serum (FBS) and incubated at 37 °C in a humidified atmosphere containing 5% CO_2_.

### 4.2. Purification of Recombinant LLO and SrtA

*Escherichia coli* BL21(DE3) with *hly* and *srtA* genes was inoculated onto fresh Luria–Bertani (LB) medium containing kanamycin at 37 °C with shaking at 200 rpm. The overnight culture was diluted 1:100 into fresh LB medium containing kanamycin and grown at 37 °C until Abs600 reached 0.6. Protein expression was induced by adding isopropyl β-D-1-thiogalactopyranoside (IPTG), and the cultures were further incubated at 18 °C with shaking at 160 rpm overnight. Cells were harvested by centrifugation at 4000 rpm for 20 min at 4 °C and resuspended in lysis buffer. After sonication on ice, the lysate was centrifuged at high speed, and the supernatant containing the soluble target proteins was collected. Protein purification was performed by nickel-nitrilotriacetic acid (Ni-NTA) [52] and analyzed using sodium dodecyl sulfate polyacrylamide gel electrophoresis (SDS-PAGE) [47]. The concentrations of the purified LLO and SrtA were 1326 μg/mL and 1656 μg/mL, respectively.

### 4.3. LM Adhesion and Invasion Assay

HeLa cells were seeded into a 24-well plate at 1 × 10^5^ cells/well and cultured overnight. The next day, fresh DMEM that contained LM (multiplicity of infection = 50) and different concentrations of ptt (0, 16, and 32 μg/mL) was used to replace the medium, and cells were incubated for 1 h. Then, cells were washed with PBS, and then 200 μL of 0.1% Triton X-100 was added to each well, followed by incubation at room temperature for 10 min to fully lyse the cells. The lysate was plated onto BHI agar and cultured overnight. For the invasion assay, cells were treated with 50 μg/mL gentamicin for 1 h to kill extracellular bacteria; all other procedures were consistent with the adhesion assay [53].

### 4.4. SrtA Activity Inhibition Assay

Purified SrtA protein (8 µg) was incubated with different concentrations of ptt at 37 °C for 30 min, followed by the addition of the substrate peptide Dabcyl-QALPETGEE-Edans (5 µg; GL Biochem, Shanghai, China). After incubation in the dark for 60 min, fluorescence signals were recorded using a microplate reader, and the inhibition of SrtA activity by ptt was analyzed based on the fluorescence value. Protease K, which cuts the substrate peptide, was used as the positive control, and the sample without the substrate peptide in the reaction system was used as the negative control. The buffer solution was: Tris-HCl at 50 mM, CaCl2 at 5 mM, and NaCl at 150 mM (pH 7.5) [54,55].

### 4.5. Computational Biology Experiments

The crystal structures of InlA, InlB, SrtA, and LLO were obtained from the Protein Data Bank (PDB) [56], with PDB IDs of 1O6S, 1D0B, 5HU4, and 4CDB, respectively. The original structures were processed by removing solvent molecules, ligands, and ions to obtain clean protein structures. The proteins were used as receptors, while ptt was selected as the ligand. Prior to molecular docking, both receptors and ligands were subjected to hydrogen addition and charge assignment.

Molecular docking was performed using AutoDock Vina version 1.2.7 [57], and the MD simulations were performed using GROMACS 2020.6. [58] The initial protein–ligand complex structure was obtained from molecular docking results. The protein was parameterized with the ff14SB force field, while the ligand was processed by antechamber and parmchk2 with the GAFF2 force field. The complex was solvated in an octahedral TIP3P water box [59] with a minimum distance of 1.0 nm from complex to box boundary, and counter-ions were added to neutralize the system. Energy minimization was performed using the steepest-descent algorithm with a step size of 0.01 nm, and the simulation converged when the maximum force was below 1000 kJ·mol^−1^·nm^−1^. Subsequently, a 500 ps NVT ensemble equilibration simulation was carried out at 300 K using the leap-frog integrator with a time step of 2 fs. After equilibration, a 100 ns unrestrained production MD simulation was conducted under NPT conditions with a time step of 2 fs. Trajectory snapshots were saved every 10 ps. The binding mechanisms between ptt and SrtA were evaluated by analyzing RMSD, protein–ligand distance, the relative position of ptt within the binding pocket, binding free energy, and residue-based energy decomposition. The predicted LM SrtA–ptt complex was structurally aligned with the reported Sa-SrtA–LPETG co-crystal structure. Amentoflavone and atractylodin were docked with LLO using the same docking protocol as described above.

### 4.6. Measurement of MIC and Growth Curves

The MIC value of ptt against LM was determined using the experimental standards of the Clinical and Laboratory Standards Institute (CLSI) [60]. First, BHI containing 1–1024 μg/mL of ptt was added to a 96-well plate. Then, the bacterial suspension of LM cultured to the log phase was added, and the final concentration in each well was 5 × 10^5^ colony-forming units per milliliter (CFUs/mL). After static incubation at 37 °C for 24 h, the lowest ptt concentration with no bacterial growth was determined as the MIC. For the growth curve assay, LM was cultured in BHI that contained various concentrations of ptt (0, 16, 32, 64, and 128 μg/mL). The initial Abs600 of LM was approximately 0.3. Samples were collected every hour to measure Abs600, and the growth curve was drawn according to the data to assess the effect of ptt on the growth of LM.

### 4.7. Real-Time Quantitative Polymerase Chain Reaction (RT-qPCR)

LM was cultured with or without ptt (32 μg/mL) at 37 °C with shaking or at 30 °C statically. Total RNA of the samples was extracted using the Trizol reagent (Sangon Biotech, Shanghai, China). Subsequently, reverse transcription was performed using the All-in-One First-Strand Synthesis MasterMix (with dsDNase) kit (Yugong Biolabs, Lianyungang, China), followed by the qPCR assay using the Universal Fast SYBR qPCR SuperMix kit (Saituobiotech, Heze, China). The relative expression levels of *inlA* and *inlB* were determined in samples cultured at 37 °C, and the critical flagella genes *fliM*, *fliY*, and *flhB* were determined in samples cultured at 30 °C. Relative gene expression was quantified by the 2^(−ΔΔCt)^ method, using *gyrB* as the reference gene. The primers used in this assay are shown in Table S1. The reaction parameters used for qPCR are shown in Table S2.

### 4.8. Hemolytic Activity Assay

Purified LLO protein was incubated with various concentrations of ptt (0, 4, 8, and 16 μg/mL) at 37 °C for 20 min, then sterile defibrinated sheep blood was added to reach a final concentration of 2.5%. Samples were centrifuged at 12,000 rpm for 2 min after incubating for another 10 min. Equal volumes of supernatant were used to detect Abs543. The culture supernatant of LM was pre-incubated statically with different concentrations of ptt (0, 8, 16, and 32 μg/mL) at room temperature, and the hemolytic activity of these samples was measured by following the same procedures. The culture supernatant of LM that was cultured with various concentrations of ptt (0, 16, and 32 μg/mL) was harvested, and the hemolytic activity of these samples was measured by following the same procedures. Sterile defibrinated sheep blood cells treated with sterile PBS or sterile water were set as the negative or positive control.

### 4.9. Western Blotting

LM was treated with ptt at concentrations of 32 μg/mL and 64 μg/mL, respectively. The bacterial culture was centrifuged at 12,000 rpm for 2 min, the supernatant was collected, and acetone was added to precipitate the proteins. SDS-PAGE loading buffer was added to the samples after the acetone was discarded. After treatment at 100 °C for 5 min, equal-volume samples were separated with 10% gels and transferred onto polyvinylidene fluoride (PVDF) membranes [61]. After blocking with 5% skim milk, the membranes were incubated with an LLO antibody, followed by an incubation with anhorseradish peroxidase (HRP)-conjugated goat anti-mouse secondary antibody. The bands were visualized, and images were obtained using a Western blot imaging system (Tanon 5200, Tanon Science Technology Co., Ltd., Shanghai, China). The band intensities were quantified using Fiji (ImageJ 1.54p). LLO protein was treated with different concentrations of ptt at 37 °C for 20 min, then sheep blood cells were added (final concentration 1%), and samples were treated on ice for 2 min. Then SDS-PAGE loading buffer without 2-mercaptoethanol was added, and samples were heated to 55 °C for 10 min. The proteins were separated by 6% gel. The subsequent procedures were performed as described above, except that the membranes were incubated with an anti-His tag antibody, followed by incubation with an HRP-conjugated goat anti-mouse secondary antibody.

### 4.10. Experiment on Inhibition and Destruction of Bacterial Biofilms

LM was co-cultured with different concentrations of ptt (0, 32, 64, and 128 μg/mL) in a 96-well cell culture plate (1 × 10^6^ CFU/well) at 37 °C for 24 h. Following this, the culture medium was discarded, and the wells were washed with sterile PBS, followed by staining with a 0.1% crystal violet solution for 30 min. Then, a 33% glacial acetic acid solution was added to solubilize the samples, and Abs570 was measured to determine the inhibitory effect of ptt on biofilm formation. To quantify viable bacteria within the biofilms, LM biofilms were grown as described above. After removing the culture medium and washing with PBS, the biofilms were resuspended, serially diluted, and plated for enumeration.

For the biofilm eradication assay, LM was inoculated into a 96-well plate and cultured at 37 °C for 24 h to form biofilms. Subsequently, the medium was discarded, and samples were washed; PBS containing different concentrations of ptt (0, 32, 64, and 128 μg/mL) was added, and samples were incubated for another 24 h to disrupt the pre-formed biofilms. The Abs570 of each sample was then measured following the same method described above.

### 4.11. Swimming Motility and Flagellar Morphology of LM

LB agar at 0.35% containing 32 μg/mL ptt was added to a 6-well plate. LM suspension (2 × 10^6^ CFU/mL) was spotted onto the center of the medium. The plate was then incubated statically at 30 °C overnight, and the colony diameter was measured to analyze the effect of ptt on LM motility. To analyze the effect of ptt on bacterial flagellar formation, LM was cultured statically at 30 °C with or without ptt (64 μg/mL). The bacteria were harvested after centrifugation (1000 rpm, 10 min [62]) and were stained with 0.5% uranyl acetate; then the flagella were observed using a TEM (JEM1400, JEOL Ltd., Tokyo, Japan).

### 4.12. Establishment of the G. mellonella Infection Model

*G. mellonella* without body spots were selected and randomly divided into three groups (10 per group). LM was injected into *G. mellonella* (2 × 10^6^ CFU/individual) using a microinjection pump. For the treatment group, each larva was treated with ptt (1.25 mg/mL) 30 min after LM injection; for the blank control and LM treatment group, each larva received a subsequent injection of 10 μL of sterile PBS. *G. mellonella* were incubated at 37 °C, and their survival status was observed every 12 h. The final survival was calculated to analyze the protective effect of ptt on the infection model. Purified LLO protein (2.65 μg/individual) was co-incubated with ptt (1.25 mg/mL) at 37 °C for 24 h, followed by injection into *G. mellonella* larvae. The LLO injection group was set as the control group, the LLO and ptt co-incubation group as the treatment group, and the PBS injection group as the blank control group. The final survival was calculated to analyze the protective effect of ptt on this infection model. Samples from different groups were stained with H&E, and the tissue damage was observed.

### 4.13. Data Analysis

Data obtained from three independent experiments are presented as means ± SDs. Statistical analyses were performed using GraphPad Prism 9.5.0. Differences between groups were analyzed using the unpaired *t*-test. For comparisons among multiple groups, one-way ANOVA followed by Dunnett’s post hoc test was applied. Survival analysis was performed using the log-rank (Mantel–Cox) test. Statistical significance was defined as * *p* < 0.05, ** *p* < 0.01, and ns, not significant.

## 5. Conclusions

Ptt is a natural compound extracted from ginseng. This study demonstrates that ptt itself does not possess direct bactericidal activity, but ptt reduces the pathogenicity of LM through a multi-target anti-virulence mechanism. Specifically, ptt significantly reduced LM adhesion and invasion into epithelial cells, potentially by interfering with the interactions between InlA/InlB and their host receptors and suppressing the transcription of *inlA* and *inlB*. Furthermore, ptt was predicted to interact with SrtA and impaired its biological function, resulting in the decreased anchoring of SrtA-dependent virulence proteins to the bacterial cell wall. Ptt also inhibited LLO oligomerization and reduced the amount of LLO secretion, thereby attenuating the hemolytic activity of both purified LLO and LM culture supernatants, which may contribute to preventing LM escape from host immune clearance. In addition, ptt suppressed flagellar formation by downregulating the transcription of key flagellar genes, leading to impaired motility. Ptt did not affect bacterial growth but significantly inhibited LM biofilm formation and disrupted pre-established mature biofilms. In vivo, ptt provided systemic protective effects against both LLO- and LM-induced toxicity in *G. mellonella* infection models. Collectively, these findings identify ptt as a promising lead compound for the development of novel anti-LM infection strategies.

## Data Availability

Data are provided within the manuscript.

## Supplementary Materials

The following supporting information can be downloaded at: https://www.mdpi.com/article/10.3390/ijms27219745/s1, Figure S1: The crystal structure of InlA and its host substrate (E-cadherin) and the residues involved in their interaction; Figure S2: The crystal structure of InlB and its host substrate (Met protein) and the residues involved in their interaction; Figure S3: The potential binding sites of the other two representative binding modes of ptt and InlA; Figure S4: Structural alignment of the LM SrtA–ptt complex with the Sa-SrtA–LPETG co-crystal structure; Figure S5: The quantitative analysis of ptt on LLO oligomerization and secretion, and SDS-PAGE image of purified LLO and SrtA protein; Figure S6: The binding mode of LLO with its various inhibitors; Table S1: The primers used for RT-qPCR; Table S2: The reaction parameters used for qPCR.
