# Supplementary material for: Protopanaxatriol Inhibits Listeria monocytogenes Infection Through a Multi-Target Virulence Suppression Strategy
Source: Int J Mol Sci. 2026 Oct 30;27(21):9745. doi: 10.3390/ijms27219745 (PMC13763453; doi:10.3390/ijms27219745)
Supplement: Supplementary file 1 [file ijms-27-09745-s001.zip › ijms-4594349-supplementary.pdf]

Supplementary files for

Protopanaxatriol inhibits *Listeria monocytogenes* infection through a multi-target virulence suppression strategy

Junlu Liu[1], Jiahui Lu[1], Lanqi Liu[1], Rui Liu[1], Biyu Lan[1], Yingxin Qi[1], Decheng Yang[1], Hao Li[1], and Guizhen Wang[1*]

[1]College of Biological and Food Engineering, Jilin Engineering Normal University, Changchun, 130052, China

*Correspondence: Guizhen Wang: wanggz@jlenu.edu.cn

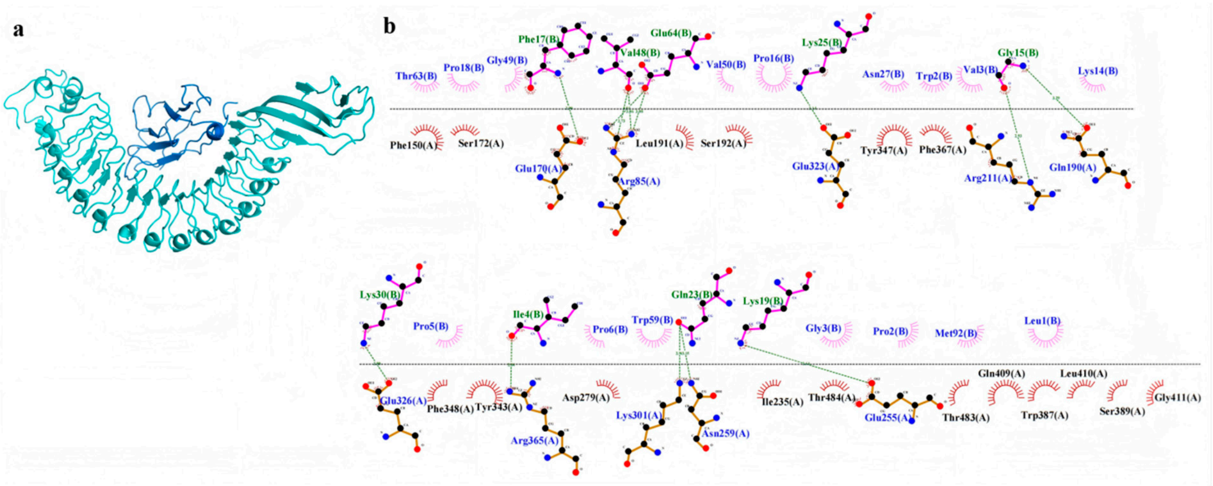

Figure S1. The crystal structure of InlA and its host substrate (E-cadherin) and the residues involved in their interaction. (a) Overall structure of the InlA-E-cadherin complex. (b) Interacting residues at the interface between InlA and E-cadherin.

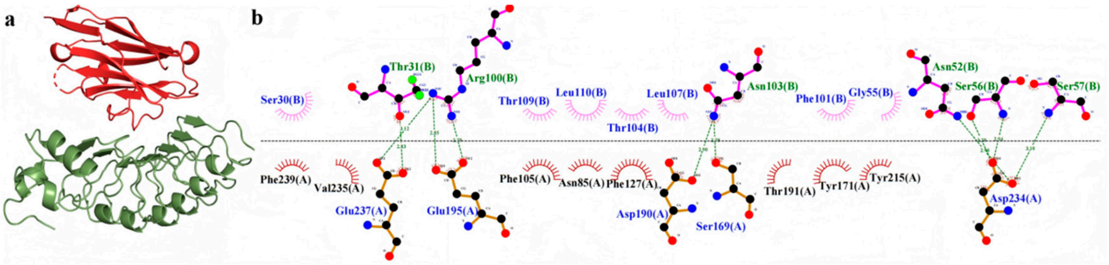

Figure S2. The crystal structure of InlB and its host substrate (Met protein) and the residues involved in their interaction. (a) Overall structure of the InlB-Met protein complex. (b) Interacting residues at the interface between InlB and Met protein.

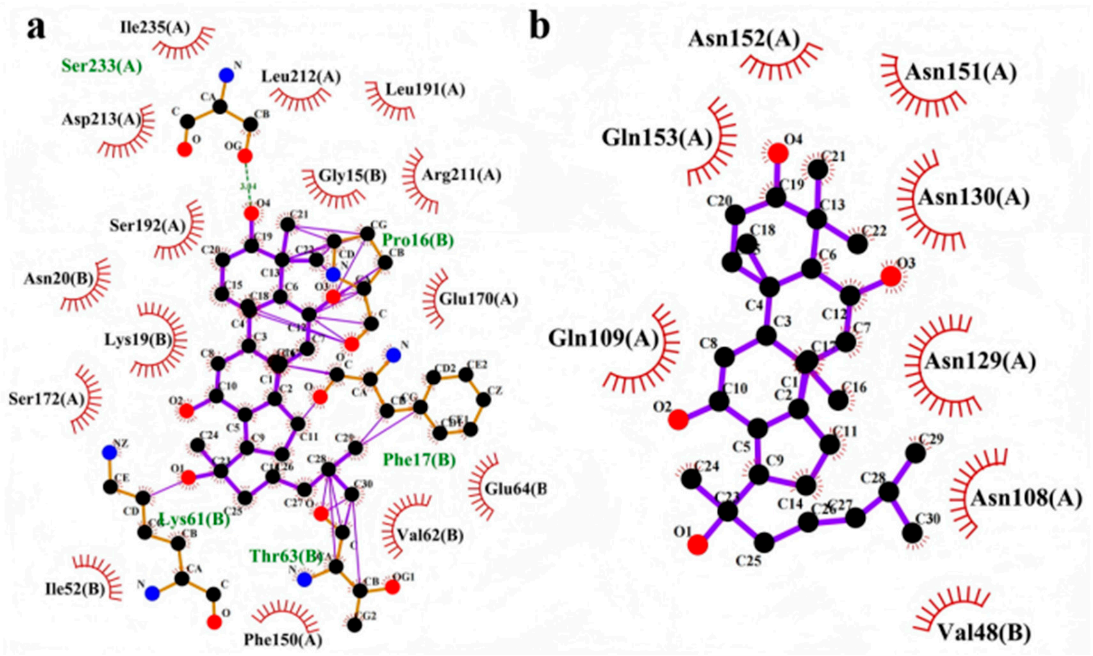

Figure S3. The potential binding sites of the other two representative binding modes of ptt and InlA. (a, b) Two representative binding modes of ptt docked at the interface between InlA and its natural substrate. Hydrogen bonds are indicated by green dashed lines, and hydrophobic interactions are shown as red arcs.

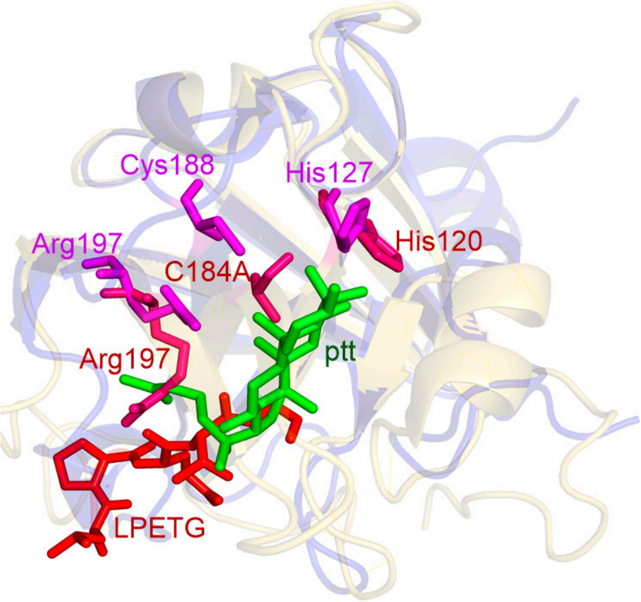

Figure S4. Structural alignment of the LM SrtA–ptt complex with the Sa-SrtA–LPETG co-crystal structure. LPETG is shown in red, ptt in green, the active site of LM SrtA in pink, and the active site of Sa-SrtA in red. Cys184 was mutated to Ala184 in the Sa-SrtA structure.

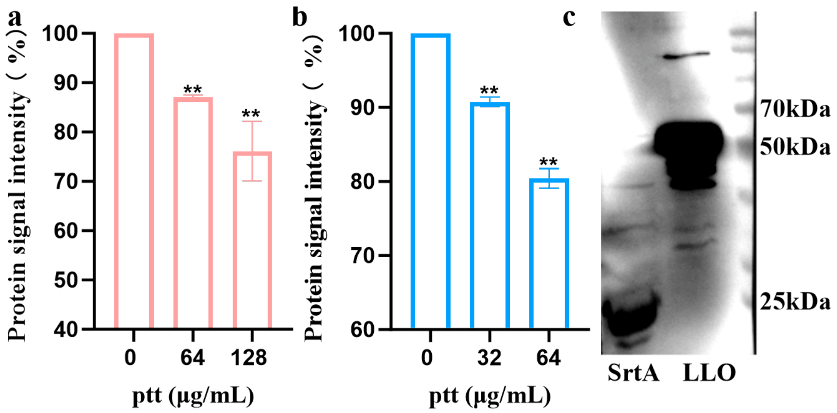

Figure S5. The quantitative analysis of ptt on LLO oligomerization and secretion, and SDS-PAGE image of purified LLO and SrtA protein. (a) Quantitative analysis of ptt inhibiting the formation of LLO oligomers. (b) Quantitative analysis of ptt reducing LLO secretion. (c) Purity of recombinant SrtA and LLO. Data are shown as meas with SD, n=3, ** $p < 0.01$.

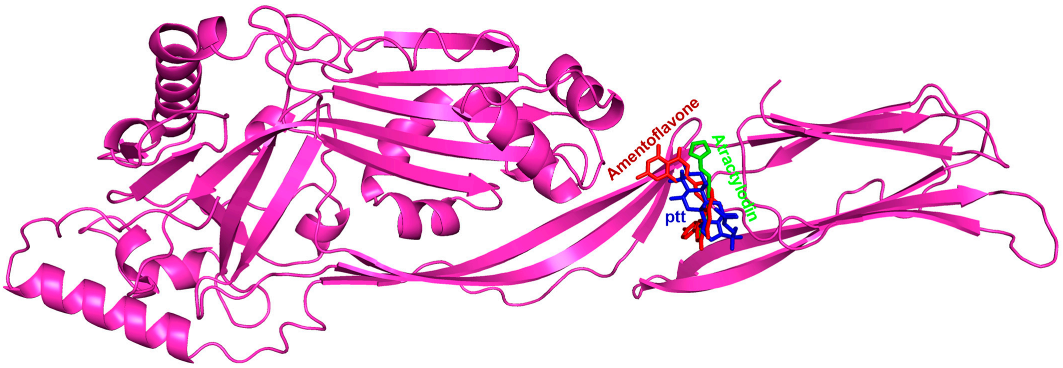

Figure S6. The binding mode of LLO with its various inhibitors. Ptt and reported LLO inhibitors bind to the same pocket. Amentoflavone and atractylodin are two reported LLO inhibitors confirmed to bind to the junction between domains 3 and 4 of LLO and inhibit its function. Based on blind docking, ptt was also found to bind to this region. Amentoflavone is shown in red, atractylodin in green, and ptt in blue.

Table S1 The primers used for RT-qPCR assay

**Primer name**	**Oligonucleotide (5'-3')**	**Length (bp)**
inlA-F	tggctttcagctgggcataa	177
inlA-R	agctggtgcaattaaagcgc	
inlB-F	cgggaatgcaggcatctaca	160
inlB-R	ttccgctttagtccagccaa	
inlC-F	gtttttccagatcccggcct	146
inlC-R	tgcattcccgcaagagattg	
hly-F	tgccaggtaacgcgagaaat	135
hly-R	tggtgccccagatggagata	
fliM-F	agcttgaagtaggcgacgtc	152
fliM-R	ctctgtccgccctgtatgac	
fliY-F	tcccgcttgaagtagaaggc	165
fliY-R	cagctggtttttccggttcg	
flhB-F	ttgaaacagcctccgaagct	128
flhB-R	tgcacgccgtagttcatcaa	
gyrB-F	ggaagaacaaggcgaaacgg	126
gyrB-R	aaagcaagttcgcgtgtacg	

Table S2 The reaction parameters used for qPCR

**Category**	**Temperature**	**Time**
	95 ℃	30 s
qPCR	95 ℃	5 s
	60 ℃	10 s
